# Whey Utilization: Sustainable Uses and Environmental Approach

**DOI:** 10.17113/ftb.59.02.21.6968

**Published:** 2021-06

**Authors:** Elizabeta Zandona, Marijana Blažić, Anet Režek Jambrak

**Affiliations:** 1 Karlovac University of Applied Sciences, Trg J.J. Strossmayera 9, 47000 Karlovac, Croatia; 2 Faculty of Food technology and Biotechnology, Pierottijeva 6, 10000 Zagreb, Croatia

**Keywords:** whey utilization, whey proteins, lactose recovery, biorefineries

## Abstract

The dairy industry produces large amounts of whey as a by- or co-product, which has led to considerable environmental problems due to its high organic matter content. Over the past decades, possibilities of more environmentally and economically efficient whey utilisation have been studied, primarily to convert unwanted end products into a valuable raw material. Sustainable whey management is mostly oriented to biotechnological and food applications for the development of value-added products such as whey powders, whey proteins, functional food and beverages, edible films and coatings, lactic acid and other biochemicals, bioplastic, biofuels and similar valuable bioproducts. This paper provides an overview of the sustainable utilization of whey and its constituents, considering new refining approaches and integrated processes to convert whey, or lactose and whey proteins to high value-added whey-based products.

## INTRODUCTION

Due to the continuous growth of the dairy industry, large quantities of by-products are produced, mainly whey. Cheese whey is a strong organic effluent that can pose a risk to the environment if not properly managed. The chemical oxygen demand (COD) of cheese whey can range from 50 to 80 g/L, while biochemical oxygen demand (BOD) varies from 40 to 60 g/L (*1*). Lactose, fat, and proteins constitute the main fraction of the organic load. In the absence of sustainable practices, whey is considered the most important environmental pollutant of the dairy industry because a large amount of whey is disposed of as wastewater and is associated with serious environmental hazards (*2*). The disposal of whey also represents a significant loss of potential nutrients and energy, so in order to utilize the nutritional value of whey and at the same time mitigate the harmful effects of disposal in the environment, it is important to direct whey management towards a cost-effective and sustainable way of utilization and directing it into the production of novel valuable products at the same time. At the UN Summit on Sustainable Development held in New York in September 2015, ’Transforming our World’, the UN Agenda 2030 for Sustainable Development (*3*) was adopted as a key global political platform for addressing many of today's challenges in their interconnected economic, social, environmental and political security dimensions. The main backbone of this ambitious development agenda is the 17 sustainable development goals (SDGs) elaborated in 169 closely related sub-goals, some of which are related to sustainable waste management of all forms of waste throughout their life cycle, reduction of emissions to the air, water and soil, and reduction of waste generation with increased share of recycling and reuse (*3*). In this sense, the attitude towards whey has changed from a waste to a valuable dairy by-product, and series of studies have been conducted to find feasible, environmentally friendly whey utilization alternatives, instead of just disposing the whey in the field (*4*). The high nutritional value of whey and its health benefits have resulted in nearly 50% of residual whey being recycled for the production of value-added products in the food and chemical industries (*5*). The traditional use of whey and its constituents in human and animal nutrition as a health promoter has been reported previously, with several biotechnological approaches and process technologies developed to convert this side-product into a source of high-value nutritional components (*6*). New methods of whey utilization are contributing to the advancement of applied technology. Ultrafiltration and nanofiltration have strengthened the utilization of whey streams, while fermentation processes for conversion of whey into high-value products have emerged as a potential pathway for biorefinery development (*7*). The simultaneous incorporation of several work units in one process is a great economical and sustainable alternative to using whey for the creation of more valuable products, thus reducing the impact of whey on the environment.

The aim of this study is to give an overview of the sustainable use of whey, as well as lactose and proteins from whey to produce high value-added products and to identify which SDGs can benefit from the reuse of dairy waste and by-products. Recent advances and new findings in refining technologies for sustainable whey management are elaborated, and environmental practices focused on reducing the ecological footprint are considered.

## WHEY COMPONENTS AND THEIR BENEFITS

Whey can be defined as the yellow-green watery part of milk (serum) that remains when the curd is separated during cheesemaking. It accounts for about 85–90% of the volume of milk and contains about 55% of the nutrients in milk. The average content of whey dry residue is: 70% lactose (depending on the acidity of the whey), 14% proteins, 9% minerals, 4% fats and 3% lactic acid (*8*). Based on the method of the milk protein coagulation, it is classified into two categories: sour and sweet whey. Sour whey, with a pH<5, is a by-product of fermentation or processes using the addition of organic or mineral acids for casein coagulation, *e.g.* the production of fresh cheese or the production of industrial casein. Sweet whey is derived from the production of cheese or certain casein products where processing is based on the coagulation of casein by rennet, an industrial coagulant containing chymosin or other casein-coagulating enzymes (proteolytic enzymes) with a pH=6-7 (*9*). It contains several value-added commodities due to its high level of fat, lactose and proteins, while sour whey contains higher levels of lactic acid, calcium and phosphorus (*10*) and is usually combined, stored and treated with plant’s primary washing waters. Although the sour whey is low in lactose and is further diluted with the wash streams, it still has high levels of COD and total organic carbon (TOC) and poses a high risk to the environment when discharged as effluent. Therefore, it should be subjected to a purification process before discharge into the receiver (*11*). In general, whey has high nutritional value and is easily digestible and assimilable. It is also considered an excellent source of functional proteins and a rich source of vitamins B, minerals (Ca, P, Na, K, Cl^−^, Fe, Cu, Zn and Mg) and lactose (*2*, *12*). Because of excellent nutritional and functional properties of whey solids, a substantial portion of whey is processed into whey powders, while the remainder is used for the production of sweet whey powder, demineralised whey, delactosed whey, whey protein concentrate (WPC), whey protein isolate (WPI) or lactose (*6*).

## SUSTAINABLE UTILIZATION OF WHEY AND ITS COMPONENTS

The dairy industry is recording a steady increase in the amount of produced whey, so new sustainable methods of whey utilization must be sought to reduce the environmental impact of whey disposal and to reduce high operating cost of whey processing. To implement sustainable whey management, a better understanding of the environmental and social impacts of products and services is needed, both of product life cycles and how they are affected by use within lifestyles. There are several directions of sustainable whey management which are mostly oriented to biotechnological and food applications for the development of value-added products such as whey powder, functional foods and beverages, lactic acid, bioethanol, bioplastics, biogas, *etc.* Large quantities of whey can be processed into bioethanol, while for smaller quantities, it is most economical to produce fermented or unfermented whey-based beverages. In that way sustainable whey management could contribute to the fulfilment of the Agenda 2030 6th SDG: Clean water and sanitation, 9th SDG: Industry, innovation and infrastructure and 12th SDG: Responsible consumption and production and its subgoals: water quality improvement, enhancement of the infrastructural sustainability, global food waste and food losses mitigation, and waste generation reduction (*3*). Defining whey as a valuable raw material and its further processing into high-value products can contribute to minimizing the release of hazardous substances to the environment and thus reduce environmental pollution. It could also halve the proportion of untreated wastewater and substantially increase its recycling and safe reuse globally. Furthermore, resource efficiency would be increased, clean and environmentally friendly technologies and industrial processes would be adopted, while global per capita waste and food losses in production and supply chains would be halved.

### Processing of liquid whey

#### Whey powders

The production of whey powders is one of the most popular ways of utilizing liquid whey. Even though drying of whey accounts for 70% of its annual processing (*12*), the development of new technologies has led to exploring alternative ways to transform whey into important value-added products. The production of whey powders usually involves several processes: a) clarification of whey, b) separation of cream and pasteurization, c) concentration of total solids (40-60% by using evaporation), d) lactose crystallization, and e) drying of whey (removal of water by spray drying). If lactose crystallization is not carried out, the solid mass of the formed bulk powder is suitable only for animal feed as an inexpensive source of high-quality proteins and carbohydrates. Implementation of different drying techniques does not produce residues that need to be treated separately, and the quality of the whey powder is preserved during transportation or further manipulation. On the other hand, it requires the high capital investment to purchase the necessary equipment and consumes a lot of energy during production. One of the disadvantages is also that whey powders have a relatively low selling price compared to whey protein concentrates (WPC). However, because of the high nutritional value, whey powder products are used in very different areas of the food industry; the most widespread use is as an additive in the production of a wide range of foods and beverages (*e.g.* infant formula, meat products, beverages, soups, sauces, toppings, creamers, nut coatings, pressed nuts, cheese-based sauces, potato chips, savoury flavours and savoury pastries, special bakery products such as pizza, biscuits and macaroni, and the manufacture of soufflés and cakes) (*13*). Whey powder can also be used as an adsorbent and as a fat and oil carrier. When producing the higher-grade whey protein powders, the whey is additionally treated by membrane separation, usually ultrafiltration or diafiltration (*8*). Foods made with the addition of whey powder may have improved sensory properties and enhanced physical characteristics (foaming or acid stability). However, such foods may also have the same texture, taste and appearance as cheese-containing foods (*6*). There are a variety of beverages with whey as the main or major ingredient, along with beverages based on whey-derived ingredients, *i.e.* WPC, WPI and whey protein hydrolysate (WPH) (*7*). These ingredients are added to beverages with high protein content, mainly sports drinks and drinks for the malnourished. Every year a small amount of WPC is produced, WPI even to a lesser extent, while the residual permeate can be recycled for lactic acid, bioethanol or lactose production (*12*).

#### Functional foods and beverages

Functional foods and beverages are one of the most ambitious and innovative categories in the food industry, continuing to generate a great deal of interest among many consumers as they offer health benefits beyond basic nutrition. Whey and its components are increasingly used as functional ingredients in dietary and health products, while bioactive proteins are more often used in both the pharmaceutical and nutritional industries (*8*). To date, researchers have focused on investigating the production of whey-based beverages from native sweet and sour whey, or from powdered, deproteinised and thinned whey (*14*). The largest dairy companies in the world have already introduced a new generation of whey-based products (*12*). Although the production of such beverages is proving to be the most economical approach to the use of whey in human nutrition, there are several difficulties regarding their production, such as susceptibility to microbial spoilage due to the high water content, and the sensitivity of whey proteins to heat treatments at temperatures above 60 °C. Most whey proteins precipitate after the usual thermal treatment of whey (at 72 °C for 15-20 s) (*2*). Therefore, much research aims to implement non-thermal techniques in the production of whey beverages, such as membrane separation, high-intensity ultrasound or supercritical carbon dioxide technology. The implementation of non-thermal methods in the production of whey beverages overcomes the above-mentioned difficulties, and improves the properties of existing products (*15*-*18*).

#### Biogas

Considering the new environmental rules, the use of digestion processes has become a quite common alternative in the treatment of agro-industrial residues. In addition, waste digestion produces biogas that can be used in power generation, resulting in both environmental and economic benefits. Because of its high organic and low buffer capacity, anaerobic digestion of whey leads to prompt acid evolution and low biogas production, so to increase the productivity whey should be mixed with other types of wastes and/or manure (*6*). Antonelli *et al.* (*19*) reported the tremendous energetic potential of biogas produced when cheese whey is digested using swine wastewater as inoculum. They noted 53.11% reduction of volatile solids and biogas yield of 270 L with 63% methane during digestion at 32 °C, and 45.76% volatile solid reduction and biogas yield of 171 L with 61% methane at 26 °C.

Waste minimization issues bring considerable attention to the development of sustainable fuels from renewable sources, also known as ’green technology’. Hydrogen is a so-called ’clean’ energy that does not generate greenhouse gasses or cause acid rain. Due to its low solubility, it is easy to hydrolyse and purify, it has high energy efficiency and can be used directly in fuel cells to generate electricity. It is a by-product of the anaerobic conversion of organic wastes by anaerobic and photosynthetic microorganisms into organic acids which are then used for methane generation (*20*). Fermentative bacteria, anaerobic bacteria and cyanobacteria are the three most common types of microorganisms that produce hydrogen. Lactose-rich wastes or by-products from the dairy industry have huge potential for biohydrogen production. Numerous operating parameters and configurations of bioreactors have been investigated in order to produce biohydrogen using cheese whey. Most studies addressed renewable feedstock processing using mono-digestion of a single substrate with a lower yield of H_2_, probably due to the poor buffering capacity of substrates and nutrient limitation (*21*). To overcome these limitations, some researchers suggested the use of co-digestion of two or more substrates for dark fermentation. Rosa *et al*. (*22*) studied the influence of inoculum sources and the co-fermentation of cheese whey with glucose. Lima *et al*. (*23*) used an anaerobic sequencing batch reactor operated with immobilized biomass on an inert support (AnSBBR) and noted that the filling time had no effect on biohydrogen production, while the feed concentration showed an optimal point for the COD of 5400 mg/L, reaching values of 0.80 mol H_2_ per mol lactose and hydrogen production of 660 mL/(L·day). They were also the first to report a strong influence of temperature on biohydrogen production, with the lowest temperature (15 °C) showing the best results (1.12 mol H_2_ per mol lactose). Rivera *et al.* (*24*) showed that microbial electrolysis cells can be considered for the treatment of cheese whey to obtain biohydrogen. Blanco *et al.* (*25*) described the anaerobic structured-bed reactor (ASTBR) as a likely alternative for fermentative biohydrogen production from cheese whey, offering better performances than conventional fixed-bed reactors. They achieved a biohydrogen yield of 2.4 mol H_2_ per mol lactose with an average yield of (1.4±0.7) mol H_2_ per mol lactose over a 32-day period.

### Lactose recovery and utilization

Lactose (4-O-β-d-galactopyranosyl-Dd-glucose) is a basic component of whey solids (70-72% total solids) and a very important energy source, making whey a potential raw material for the production of lactose as well as whey-based value-added products. From a health and nutritional point of view, lactose presents many benefits because it acts like dietary fibre and has prebiotic properties. In that manner, lactose facilitates the intestinal absorption of various minerals such as calcium, phosphorus and magnesium (*26*). It is also used as a nutrient source and substrate for the production of lactic acid and short carbon cycle fatty acids (SCFA) by intestinal bacteria, thereby establishing a mildly acidic reaction in the intestine and preventing the growth and proliferation of harmful bacteria (*27*). Also, it has a lower impact on blood sugar levels due to the low glycaemic index (half that of glucose) (*28*). Lactose may be recovered from whey by isolation from deproteinised whey (*e.g.* whey permeate obtained by ultrafiltration) using several methods: concentration of whey by evaporation, crystallization of lactose from concentrated whey (*29*) and separation of the obtained crystals by centrifuge or decanter (*13*). Lactose is the main component causing high BOD and COD levels in whey, so its recovery could reduce the BOD value by more than 80% (*30*). In this regard, lactose recovery could solve waste utilization problems and environmental issues, respectively. Depending on its quality, the recovered lactose may further be supplied to the food, pharmaceutical, dairy and beverage (*e.g.* food-grade or pharmaceutical-grade) industries. Generally, it is used in the food and confectionery industries, above all in the baking industry as a crust browning promoter, and in the pharmaceutical industry as an excipient. Further, it is possible to produce new whey-based products by the degradation of lactose by microorganisms. Various biotechnological processes have been developed to recover lactose from whey and its further processing into products of industrial importance, *e.g.* organic acids and alcohols, such as lactic and citric acids, ethyl alcohol, kefir-like fermented whey beverages, single cell proteins, probiotic starter cultures, biogas, bioplastic and ethyl lactate (*31*).

#### Lactic acid

Lactic acid (LA; 2-hydroxypropanoic acid) is a promising platform chemical existing as two isomers, l(+) and d(-). Both of them can be produced biotechnologically, but only l(+) isomer can be used in the food industry, because d(-) is harmful to humans (*32*). In contrary, d(-) has a range of applications in the production of polylactic acid-based polymers (PLA), can be converted into several chemicals of industrial importance such as pyruvic acid, acrylic acid, 1,2-propanediol and lactate ester (*33*). LA and its derivatives have long been applied in food, pharmaceutical, textile, leather and chemical industries, primarily as preservatives and acidifiers. Lately, its production increased due to the application in the production of the environmentally friendly biodegradable polymers (PLA) (*6*) with the intention of replacing significant amounts of petroleum-based plastics and contributing to climate change mitigation goals. Lactose can be effectively converted to lactic acid by fermentation using bacteria, fungi and yeast (*34*). It is a fermentation product of a wide group of microorganisms (*35*), *e.g. Lactobacillus, Bacillus*, *Enterococcus*, *Lactococcus*, *Pediococcus*, *Streptococcus* and *Candida*, and filamentous fungi *Rhizopus oryzae* (*33*). Only few of them are used by the industry starting with native LA producers *Lactobacillus delbrueckii* and *Sporolactobacillus*, recombinant organism *Escherichia coli*, *Bacillus coagulans*, *Corynebacterium glutamicum* and *B*. *licheniformis* (*36*). *Kluyveromyces marxianus* var. *marxianus* has also been successfully used for LA production (*13*). LA is primary synthetised from pure and expensive lactose, glucose or sucrose. Because of the high prices of pure raw materials, research is directed towards more economically and environmentally friendlier approaches, *e.g.* obtaining LA from waste effluents, such as whey (*37*). Whey needs to be pretreated by membrane techniques prior to the production of LA to reduce its protein content and increase the concentration of lactose and mineral salts. Therefore, precipitating whey proteins at a high temperature, and then using the remaining lactose as the carbon source for fermentation with some other nitrogen supplement such as yeast extract represents the main LA production method (*38*). When utilizing a complex feedstock such as whey, which contains a mixture of carbohydrates, fermentation using mixed cultures is recommended to provide the desirable combinations of metabolic pathways for better carbohydrate conversion into LA (*39*). Lactic acid bacteria have limited ability to synthesize amino acids and vitamins B, so nutrient supplementation is a key factor limiting the process efficiency, especially in industrial scale application. In that manner, carbohydrates from whey are metabolised with the addition of nutrients such as yeast extract, peptone, or corn steep liquor as nitrogen sources (*40*). Liu *et al.* (*38*) established a simple and economic d-lactic acid production from cheese whey powder by employing *Lactobacillus bulgaricus* CGMCC 1.6970 and obtained a high d-lactic acid titre and productivity of 113.18 g/L and 2.36 g/(L·h), respectively. To provide an additional source of nitrogen and replace the yeast extract to a large extent, they hydrolysed the CW domain containing proteins by proteases prior to the fermentation. Reduced nutrient supplementation and more efficient and sustainable LA production can be achieved by using genetically or metabolically modified strains with new or improved properties or by cocultivation with strains that provide important nutrients. Zhang and Vadlani (*41*) used a co-culture of *L. brevis* ATCC 367 and *L. plantarum* ATCC 21028 strains for LA production from biomass-derived sugars, which increased the LA yield significantly (from 0.52 to 0.80 g/g), with a lower by-product accumulation. Sahoo and Jayaraman (*42*) described d-LA production from whey permeate by *Lactobacillus delbrueckii* and engineered *Lactococcus lactis* co-culture and also noted the enhancement in the yield of d-LA. To improve the titre and productivity of d-LA, the authors pointed out the need for fed-batch process design. Further performance improvement of the d-LA production process could be accomplished by upgrading the repeated batch and fed-batch fermentation to continuous mode (*43*), including cell recycling or by implementing the immobilized cells in fixed or fluidized bed bioreactors (Table 1) (*37*). Ziadi *et al.* (*50*) developed kinetic modelling of biomass and lactic acid production by *Enterococcus faecalis* SLT13 during batch culture in M17 and hydrolysed cheese whey in 2-L and 20-litre bioreactors.

**Table 1:** Lactic acid production using cheese whey-based medium

Substrate	Microorganism	Fermentation type	Reference
Cheese whey	*Lacticaseibacillus casei* BL23 *L. casei* BL71 (BL23 ccpA::erm)	Stirred tank batch fermentation	(*44*)
Cheese whey	Polyvinyl alcohol (PVA) immobilized *Lactobacillus plantarum*	Batch and continuous fermentation	(*45*)
Acid whey	*Bacillus coagulans* A534	Batch fermentation and continuous fermentation	(*46*)
Whey and yeast extract	Immobilized mixed culture of lactic acid bacteria (*Lactobacillus acidophilus* LA-5, *Bifidobacterium* BB-12 and *Streptococcus thermophiles*)	Batch fermentation in Erlenmeyer flask	(*47*)
Whey permeate	Co-culture of *Lactobacillus delbrueckii* and engineered *Lactococcus lactis*	Batch fermentation	(*48*)
Cheese whey	Mixed cultures (the anaerobic digestate with inhibited methanogenic species)	Dark fermentation; repeated-batch mode aimed at semi-continuous lactic acid production	(*49*)

The use of whey and similar complex substrates for LA production results in a series of impurities in the fermentation broth. Therefore, to obtain pure LA, large-scale downstream processing is required, which includes several membrane separation processes (*e.g.* micro- and nanofiltration, electrodialysis with monopolar and bipolar membranes or concentration by water evaporation) (*33*). Immobilisation technology and cell recycling may improve the process by increasing cell density in the bioreactors and facilitate purification in downstream process, especially in continuous systems.

#### Bioplastic

The interlinkage of biotechnology processes is a key strategy in maximizing the use of agro-industrial wastes and increasing the potential revenue of the entire bioprocessing chain in the production of bioplastics (Table 2). Using cheese whey as a substrate for bioplastic production has lately come in focus, as the lactose present in whey permeate can be easily converted into polyhydroxyalkanoates (PHAs) and polylactic acid (PLA) (*55*). The bioplastics thus produced can be further used in the packaging, spraying materials, device materials, electronic products, agricultural products, automation products, chemical media and solvent industries.

**Table 2:** Cheese whey application in production of biopolymers

Substrate	Microorganism	Biopolymer	Reference
Cheese whey	*Lactobacillus* sp. *Rhodovulum sulfidophilum* DSM-1374	Poly(3-hydroxybutyrate)	(*51*)
Ricotta cheese exhausted whey	β-Galactosidase treatment and *Haloferax mediterranei* DSM1411	Poly(3-hydroxybutyrate-co-3-hydroxyvalerate) (PHBV) with hydroxyvalerate (HV)	(*52*)
Fermented cheese whey	Mixed photosynthetic consortium of bacteria and algae	PHA with a hydroxyvalerate (HV)	(*53*)
Sweet whey powder	Mixed microbial culture (mostly *Thauera* and the *Lampropedia* genera)	PHA	(*54*)

### Polyhydroxyalkanoates

Polyhydroxyalkanoates (PHAs) are biopolyesters synthesised as carbon and energy reserve by aerobic bacteria and accumulated as intracellular granules under growth-limiting and carbon excess conditions. PHAs include polyhydroxybutyric acid (PHB), polyhydroxyvaleric acid (PHV), 3-hydroxy-2-methylbutyrate (3H2MB) and 3-hydroxy-2-methylvalerate (3H2MV) (*40*). PHAs have mechanical and physical properties comparable to polyethylene (PE) and polypropylene (PP) but are different in their biocompatibility, biodegradability by soil bacteria, UV-resistance and oxygen impermeability (*56*). The production costs are much higher than that of conventional plastics. Therefore, to commercialize PHAs, it is necessary to reduce the production cost through the development of efficient bacterial strains, more robust fermentation/recovery processes and find suitable and cheap substrates. Chen and Patel (*57*) reported that whey compounds are suitable for biological process taking place in PHA production, and they gave an excellent review covering this subject. In the last two decades, a noticeable number of studies have been made of PHA production from whey permeate using pure cultures of wild type microorganisms or recombinant ones. Three possible routes from whey lactose to PHA emerged: direct conversion of lactose to PHA, conversion of glucose and galactose to PHA after the hydrolysis of lactose (chemically or enzymatically), and lactose fermentation to lactic acid followed by conversion of lactic acid to PHA. A limited number of microorganisms have the ability to directly convert lactose to PHAs (*e.g. Hydrogenophaga pseudoflava* and recombinant *Escherichia coli*, due to their β-galactosidase activity), so in several of these works, whey lactose was hydrolysed by lactase and used by microorganisms such as *Pseudomonas hydrogenovora* or *Haloferax mediterranei* to produce PHAs (*58*). The other way around, genetic information for PHA accumulation can be transferred from the acknowledged PHA-producers to easily cultivated non-PHA-producers, or PHA-producers can be genetically and metabolically modified to convert additional substrates to PHB to enhance conversion, optimise polyester composition, and increase productivity. Also, genetic engineering may increase the molar fraction of 3-hydroxyvalerate (3HV) in co-polyesters as demonstrated by Heinrich *et al.* (*59*).

### Polylactic acid

Polylactic acid (PLA) is a biodegradable biopolyester made by condensation of lactic acid (LA) monomers, and one of the most promising environmentally friendly (green) plastics of the era, as it closely resembles polystyrene (PS) and polypropylene (PP) in most of its properties. Due to its low toxicity, PLA has a GRAS status (Generally Regarded as Safe) and can be also used in food packaging. It is biodegradable, so it can be composted in earthen trenches along with other biodegradable materials, such as plant and vegetable wastes and animal wastes, and its disposal will not cause any environmental concern (*37*). Despite its biodegradability, if improperly disposed in landfills, it will last for years like petrol plastics. PLA occurs in three different forms: poly(l-lactic acid) (PLLA), poly(d-lactic acid) (PDLA) and poly(dl-lactic acid) (PDLLA). Even though PLLA is suitable for industrial use, its application is limited by its low thermal stability (melting point 180 °C), unlike the stereocomplexes (SC) of PLLA and PDLA, which are heat stable (melting point 230 °C), resistant to hydrolysis, and have better mechanical properties (*38*). Production of PLA increased the demand for d-LA, so an eco-friendly microbial production of d-LA came in focus. It can be produced by wild-type strains such as *Sporolactobacillus laevolacticus*, *Lactobacillus plantarum*, *Sporolactobacillus ilulins* and *Lactobacillus bulgaricus* (*36*). PLA is a good substitution for hydrocarbon-based polymers, but due to the high cost of d-LA fermentation, its production is not a competitive solution. Utilisation of renewable materials, primarily agro-industrial wastes such as whey and whey permeate is an auspicious alternative for the cost-reduced production of d-LA. Cellulac (*60*) was the first company worldwide to carry out a continuous industrial level production of lactic acid from deproteinised lactose whey. Their system used non-GMO (genetically modified organism) *Lactobacillus* (whole cells) to transform the lactose from deproteinised lactose whey into d-LA suitable for conversion to bioplastics. Prasad *et al.* (*61*) in their study demonstrated homo-lactic fermentative conversion of whey permeate by *L. lactis* to high value-added d-LA, as well as Liu *et al.* (*38*), who obtained a high d-LA titre and productivity by *L. bulgaricus* fermentation.

#### Bioethanol

Bioethanol has stood out as a potential alternative and environmentally friendly fuel for the future (green fuel). Since it does not produce any toxic emissions on combustion, bioethanol proves to be effective in decreasing air pollution and reducing global warming. Therefore, its production is empowered by legislative incentives all over the world. In 2007, more than 95% ethanol acquired in the USA was produced from corn, and the excess 5% came from wheat and barley or agro-industrial wastes (cheese whey and certain beverage remains) (*62*). To forestall the lack of food crops or rural assets, just as to mitigate the environmental impact of industrial and agricultural wastes, various strategies for bioethanol production have been developed based on implementing non-food agriculture crops and various agro-industrial wastes. Among these wastes, whey has stood out as a suitable substrate for bioethanol production due to its high organic load and high pollution potential. Although the conversion of the lactose and other whey constituents into bioethanol is barely competitive with the current technologies using sugarcane, corn starch or utilizing lignocellulosic biomass as raw material, bioconversion of whey into ethanol draws attention. Direct fermentation of whey is not economically reasonable due to the low lactose content and low bioethanol yield (2–3%), leading to high capital investments in the distillery. Better bioethanol yield may be achieved by fermenting whey with high lactose concentration, *i.e.* whey concentrated by ultrafiltration and/or reverse osmosis. The conventional industrial strain of *Saccharomyces cerevisiae* lacks lactose breakdown enzymes, so lactose has to be enzymatically hydrolysed prior to the alcoholic fermentation by *Saccharomyces cerevisiae* (*62*). In contrast, *Kluyveromyces marxianus* strains have the ability to metabolize lactose and are commonly used yeast strains for the fermentation of lactose into bioethanol. Gabardo *et al.* (*63*) stated that continuous fermentation, when cells of *K. marxianus* are immobilised in calcium alginate, improves ethanol yield, achieving 6.97 g/(L·h) productivity. Sampaio *et al.* (*64*) investigated the ability of *K. lactis* to ferment cheese whey and obtained 15.0 g/L ethanol, 0.47 g/g yield on consumed lactose and 0.31 g/(L·h) productivity, corresponding to 87.4% fermentation efficiency. Since the introduction of modern approaches such as genetic engineering, the use of engineered *S. cerevisiae* for lactose fermentation has drawn much attention and attempts have been made to construct lactose-consuming *S. cerevisiae* strains. In the commercial production of bioethanol from whey permeate, *K. marxianus* var. *marxianus* and *K. fragilis* var. *marxianus* are commonly used (Table 3), and there are some examples of such industrial units in Ireland, New Zealand, Denmark and the USA (*62*). Bioethanol obtained from whey can be utilized in food, chemical, drug and cosmetic industries and as an alternative and sustainable fuel.

**Table 3:** Bioethanol production from cheese whey

Substrate	Microorganism/enzyme source	Reference
Crude whey	Genetically engineered *S.cerevisiae* strain	(*65*)	
Crude whey	*K. marxianus* ETP87	(*66*)	
Cheese whey permeate	*K. lactis* CBS2359	(*64*)	
Cheese whey	*S. fragilis* IZ 275	(*67*)	
Cheese whey powder	*K. marxianus* and *S. cerevisiae*	(*68*)	
Whey permeate	*K. marxianus* URM 7404	(*69*)	
Cheese whey powder	*E. coli*	(*70*)	
Delactosed whey permeate	*C. glutamicum*	(*71*)	
Cheese whey powder	*K. lactis* β-galactosidase and *S. cerevisiae*	(*72*)	
Mozzarella cheese whey and sugarcane molasses	*C. tropicalis* and *B. capitatus*	(*73*)	
Cheese whey permeate	*K. marxianus* NCIM 3217	(*74*)	
Whey permeate	*L. lactis*	(*75*)	

#### Single cell proteins

Production of single cell protein (SCP) is one of basic steps in solving the problem of increasing demands for innovative and alternative food sources. SCP is defined as a ’protein extracted from cultivated microbial biomass’, referring to dehydrated cells of various microorganisms (algae, actinomycetes, bacteria, yeast, moulds and higher fungi) grown in large-scale culture systems for use as protein source in human food or animal feed. It can be used for protein supplementation as an alternative to expensive conventional sources such as soy meat and fish meat (*6*). Whey utilization as a substrate for the production of SCP may reduce polluting potential of whey and result in the production of a value-added product. Whole whey or whey permeate is a convenient substrate for the SCP production *via* direct use of lactose by lactose-consuming microorganisms, or indirectly after the hydrolysis of lactose by enzymatic or chemical means for a microorganism that does not grow on lactose. The *Kluyveromyces* species have been most widely studied for SCP production from whey, in particular *K. marxianus* or *K. fragilis* strains, which are GRAS microorganisms and offer advantages of good growth yields (*76*). Sampaio *et al.* (*77*) suggested that cheese whey, adequately enriched with salts and yeast extract, could be interestingly exploited as an alternative medium for the production of *K. lactis* as a single cell protein. Nayeem *et al.* (*78*) also proposed whey as a suitable substrate for SCP production after the fermentation using strains of *K. marxianus* where the biomass yield of 36 mg/mL was obtained with 83.33% crude protein content. Not many studies have looked at SCP microalgae. Putri *et al.* (*79*) utilized cheese whey waste as a nitrogen source for the growth of *Chlorella* sp. as a unicellular producer with high growth rate, high protein content, high chlorophyll content and low nucleic acid content.

### Whey protein utilization

Whey proteins constitute about 20% of the total proteins present in the milk. They are a mix of globular proteins with a relatively even distribution of non‐polar, polar and charged amino acids that can be isolated from whey and represent one of the nutritionally most valuable components of whey. They are composed mainly of β‐lactoglobulin (β‐Lg), α‐lactalbumin (α‐La), bovine serum albumin (BSA), immunoglobulin (Ig), thermostable proteose‐peptones and lactoferrin (Lf) representing about 50, 20-25, 10-15, 6, 1 and less than 1% of whey protein fractions respectively (*8*). Unlike caseins that exist as a micellar suspension, whey proteins have a compact globular structure with quite different amino acid profiles. Because of the smaller fraction of Glu and Pro and greater Cys/Met ratio, these proteins have a higher biological value and are more easily digested than other proteins of animal origin (*13*). They have been reported to possess a plethora of nutritional and biological benefits, which are mainly associated with the bioactive peptides that stem from proteolytic breakdown of whey proteins. Such bioactive peptides have an indispensable role in the dietary management of chronic diseases (cardiovascular, digestive, immune and nervous systems). The beneficial health effects of whey proteins can be classified as anti‐microbial, anti‐oxidative, anti‐thrombotic, anti‐hypertensive or immunomodulatory (*80*). The development of methods for protein separation, purification and drying (membrane separation and chromatography, electrodialysis, spray and freeze drying) have drawn the attention of the academic community towards the distinct biological and functional characteristics of whey proteins and widened their application (*81*). Besides food and beverages, whey proteins have other numerous praiseworthy applications in food industry, as they can be easily shaped into different bases and matrices (macro-, micro- and nano-structures) suitable for carrying several types of bioactive compounds, different flavours or compounds with high nutritional value. Also, there are several reports that indicate a targeted application of whey proteins as surface-active components, texture modifiers, foaming and gelling agents, thickening agents and emulsifiers (*82*-*84*). Recent developments in this field are focused on development of novel whey protein-based value-added products such as whey protein-based edible films, hydrogels, nanoparticles and microencapsulated products (*81*).

#### Edible films and coatings

The demand for so called ’green’ packaging has accelerated research on active bio-based packaging. Edible or biodegradable films are green alternatives to traditional plastics and thus also help in controlling environmental pollution. These biofilms have plenty of advantages, as they may replace or reinforce existing natural layers, preserve moisture and prevent loss of important components (*e.g.* flavours) and above all, can be eaten together with the product without prior removal (*85*). Reuse and recycling of agro-industrial wastes have attracted significant scientific interest towards the exploitation of whey protein in order to create edible films and coatings (Table 4). Whey protein films with their admirable oxygen barrier properties stand out as sustainable biodegradable alternative materials to replace typically used nylon or polyester films. Moreover, whey proteins can form clear films and coatings with improved mechanical and barrier properties compared to polysaccharide-based films and may provide surface sterility (*94*). Besides the improved barrier properties, such films and coating also biodegrade fast. However, to develop new eco-efficient food packaging products with improved resistance to moisture transfer and enhanced flexibility, whey proteins need to be blended with suitable plasticisers, such as sorbitol or glycerol (*94*).

**Table 4:** Whey-based edible films and coatings

Film forming material	Plasticizer	Supplement	Reference
WPI (5% *m*/*V*) and water soluble derivative of chitosan (WSCh) (3% *m*/*V*)	Glycerol (1.8% *m*/*V*)		(*86*)
WPI (*w*=8%)	Glycerol (*w*=26-54%)	Lemon and bergamot essential oils	(*87*)
WPC (*w*=10%), guar gum (*w*=0.7%)	Glycerol *(w*=5%)	*L. buchneri* UTAD104, *L. casei* UM3 (*w*=10, 20 or 30%)	(*88*)
WPI (5% *m*/*V*), wax and antifoam	Glycerol (2% *m*/*V*)	Nisin (10.000 IU); sodium benzoate and potassium sorbate (4% *m*/*V*); thyme, rosemary, basil, pimento and coriander essential oils (4% *m*/*V*)	(*89*)
WPI, chitosan	Glycerol		(*90*)
WPI (5% *m*/*V*)	Glycerol (4% *m*/*V*) Glycerol (4% *m*/*V*) and trehalose (3% *m/V*)		(*91*)
Whey protein	Water, glycerol, natural rubber latex		(*92*)
WPI, natural latex and albumin	Water		(*93*)

WPI=whey protein isolate, WPC=whey protein concentrate

Pereira *et al.* (*95*) proposed alternative biomaterials for potential uses in food applications in form of WPC nanocomposites activated with lycopene and montmorillonite nanoparticles. Qazanfarzadeh and Kadivar (*96*) reinforced WPI nanocomposite film properties with oat husk nanocellulose. Incorporation of biocompounds with natural antioxidant or antimicrobial activity into the whey protein isolate matrix has also become a field of research. A recent study by Boyacı *et al.* (*97*) describes the development of an antimicrobial film composed of whey proteins, beeswax, oleic acid and lysozyme for the protection of unpackaged food in households. Extended shelf life, as well as improved quality and safety control of the products, are among the main advantages reported using such complex films. Andrade *et al.* (*98*) investigated biodegradable whey protein-based films incorporated with ethanolic rosemary and thyme extracts, and developed an active film with 1% incorporated rosemary extract. The film presented antimicrobial activity against *L. monocytogenes* and *S. aureus*. Soukoulis *et al.* (*99*) examined the survivability of *L. rhamnosus* GG on WPC and several other biopolymers as a carrier. The use of whey proteins as a carrier (base or matrix) during incorporation of functional ingredients (such as good bacteria, probiotics and prebiotics) into novel value-added products could improve their survival rates or activity during storage and consumption.

#### Hydrogels

Hydrogels are defined as polymeric three-dimensional networks that can assimilate high amounts of water or biological fluids due to the presence of hydrophilic groups, such as –OH, -CONH, -CONH_2_- and -SO_3_H (*81*). Because of their interesting capability of consolidating the qualities of a hydrogel system with a nanoparticle, hydrogel nanoparticles have been the centre of attention in recent examination of drug delivery systems (*85*). Besides forming biofilms, whey proteins can also form polymeric 3D networks like hydrogels (*7*). In recent years, whey protein nanostructured particles have also attracted much attention because of their specific functional attributes such as: surface activity, ability to form hydrogels, GRAS properties, easy preparation, relatively low cost, and effectively monitored size distribution. To formulate novel food products, it is important to comprehend the interactions between the whey proteins and biopolymers such as pectin, κ-carrageenan, xanthan and basil seed gum (*100*, *101*). As hydrogels have specific structural and sensory characteristics for targeted functional foods, when developing new products such as fermented dairy beverages, ice cream, confectionery and bakery products, researchers are increasingly turning to hydrogel applications (*7*). Nourbakhsh *et al.* (*102*) managed to apply WPHs for encapsulation of water-soluble nutraceuticals. Su *et al.* (*103*) employed whey proteins for microencapsulation by using high internal phase emulsions stabilized with WPI microgels to enhance the viability of *Lactobacillus plantarum* as probiotics. Fang *et al.* (*104*) used WPI nanoparticles for α-tocopherol and resveratrol encapsulation as protein-based carriers for hydrophobic components. When micro- and nanoparticles are used as carriers of bioactive compounds, the controlled release of incorporated bioactive compounds is enabled during consumption, which is crucial for improvement of nutritional and functional aspects of food. Defining the nanostructure of whey protein as a GRAS material allows their wider application in food research (*105*).

## ENVIRONMENTAL APPROACHES FOR WHEY UTILIZATION

Dairy sector has a heavy ecological footprint on the earth’s ecosystems and public pressure forces the dairy industry to re-evaluate their whey management. Europe is the overall leader in dairy processing and subsequently the largest whey producer. The cheese industry produces about 115 million tonnes of whey annually and 47% of it is being directly disposed in the drains, causing serious environmental pollution problems (*12*). Owing to its high BOD and COD, whey is considered a major pollutant by-product worldwide. The contamination capability of whey has driven countries such as USA, Canada, Australia, New Zealand and the European Union to introduce strict environment protection legislation against inappropriate disposal of whey and in favour of its sustainable utilization. In Europe, the landfill disposal of cheese whey has also been abolished due to the recently developed markets for whey proteins and regulatory requirements based upon the EU Landfill Directive 1999/31/EC (*12*). These strict legislations encouraged dairy industry to explore other approaches and opportunities for the management of dairy effluents. The reduction of dairy environmental impact is possible by following correctly sustainable procedures at all steps of the dairy supply chain, also focusing on waste reduction and virtuous use of by-products such as whey. Life cycle assessment (LCA) emerged as a generally accepted approach, implying an environmental study focusing on the complete life cycle of a product or a service, from resource extraction to end-of-life products considering all steps in between, by quantifying the environmental impact, such as climate change, ecosystem quality (*i.e.* aquatic acidification and eutrophication), human health and resources (energy and water) (*106*). LCA consists of four main phases: (*i*) defining the goal and scope of an LCA, (*ii*) analysis of the inventory necessary for carrying out the study, (*iii*) calculation and assessment of environmental impacts, and (*iv*) interpretation of the results (*107*). For the dairy sector, an LCA has to consider farm production (fodder, cow raising, milking or at farm refrigeration), packaging, dairy processing (processing for production of various dairy products), distribution (transports and retailers), use phase and end-of life (*6*).

The development of integrated biorefinery concept has drawn significant attention during the last years to reduce whey disposal by introducing holistic approaches of its valorisation to formulate a plethora of end-products (*7*). The use of all raw material flows in accordance with the concept of circular economy and zero waste generation, embracing all pillars of sustainability, *i.e.* the environment, society, and the economy, is one of the main premises of biorefineries (*108*). Therefore, new refining approaches have been proposed for conversion of dairy by-products into valuable bio-based products (*e.g.* additives, bioplastics or biochemicals), even into larger quantities of less-valuable products such as bioethanol, or smaller quantities of highly priced products such as nutraceuticals (*109*). Németh and Kaleta (*11*) set up a biorefinery concept for conversion of the lactose from whey into yeast biomass for ergosterol production (*i.e.* previtamin D2). After the ergosterol extraction, the residual yeast debris was combined with the lactic acid containing residual organic part of the whey and used in propionic acid and vitamin B12 production. Pasotti *et al.* (*110*) first reported efficient ethanol production from the lactose contained in whey permeate with engineered *E. coli*. Zhou *et al.* (*72*) first proposed two-step bioprocess using *Saccharomyces cerevisiae* and *Gluconobacter oxydans* to bioconvert cheese whey into ethanol and galactonic acid. Overall, a lot of effort has been invested into developing integral processes and setting up a closed food supply chain through the manufacture of novel food products. Reported studies regarding the biorefinery concept did not cover all aspects of cheese whey utilization. Novel approaches, implementing both lactose and protein streams, will yield alternative bio-based components resulting in high value-added products with enhanced physicochemical, sensory and nutritional properties.

## CONCLUSIONS

Environmental issues have forced governments to legislate the disposal of whey, and as a consequence encouraged dairy industry to explore other approaches and opportunities for the management of dairy wastes. Due to its high polluting capacity, reuse and recycling of whey has become a great scientific challenge in order to reduce dairy wastes and meet the Agenda 2030 sustainable development goals (SDG). These scientific efforts resulted in developing different sustainable methods for dealing with the whey and changed the attitude towards whey from cheesemaking waste to value-added raw material. These methods also contributed to the fulfilment of the Agenda 2030 6th SDG: clean water and sanitation, 9th SDG: industry, innovation and infrastructure, and 12th SDG: responsible consumption and production. Whey components make whey a great base for the creation of a series of new products or an ideal alternative compound to more traditional ones. The review highlights possibilities of utilization of whey and its components, considering new refining approaches to convert dairy by-products into several valuable bio-based products. Large volumes of whey still need to be processed, so more efficient and economic integrated processes and systems must be developed, especially in the emerging fields of biochemicals, biofuels and bioplastics.
